# Can a Continuous Wound Infiltration System Replace Intravenous Patient-Controlled Analgesia for Postoperative Pain Management after a Single-Port Access Laparoscopy?

**DOI:** 10.3390/jcm13195718

**Published:** 2024-09-25

**Authors:** Jun-Hyeok Kang, Yumi Seo, Hyunji Lee, Woo Young Kim, E Sun Paik

**Affiliations:** 1 Department of Obstetrics and Gynecology, Uijeongbu Eulji Medical Center, Eulji University School of Medicine, Uijeoungbu-si 11759, Republic of Korea; junhyuck1985@gmail.com; 2 Department of Obstetrics and Gynecology, Kangbuk Samsung Hospital, Sungkyunkwan University School of Medicine, Seoul 03181, Republic of Korea; yumi4808@gmail.com (Y.S.); hjleeim@gmail.com (H.L.)

**Keywords:** single-port access laparoscopy, postoperative pain, continuous wound infiltration, intravenous patient-controlled analgesia

## Abstract

**Background:** The aim of this study was to determine whether continuous wound infiltration (CWI) can replace intravenous patient-controlled analgesia (IV PCA) and to investigate effective pain control strategies after a single-port access (SPA) laparoscopy for adnexal disease. **Methods**: A total of 470 patients (the CWI group [n = 109], the IV PCA group [n = 198], and the combined group [n = 163]) who underwent an SPA adnexal laparoscopy and who received CWI or IV PCA for postoperative pain management were retrospectively reviewed. The numeric rating scale (NRS) pain score at 6, 12, 24, and 48 h (h) after surgery and the total amount of fentanyl administered via IV PCA were collected. The incidence of postoperative nausea and vomiting (PONV) and the total amount of rescue antiemetic drugs administered were also evaluated. **Results**: The mean NRS pain scores at 6 h (combined vs. PCA vs. CWI, 3.08 vs. 3.44 vs. 3.96, *p* < 0.001), 12 h (2.10 vs. 2.65 vs. 2.82, *p* < 0.001), and 24 h (1.71 vs. 2.01 vs. 2.12, *p* < 0.001) after surgery were significantly lower in the combined group. CWI showed a similar pain-reduction effect after surgery compared to IV PCA, except for the acute phase (within 6 h after surgery). The incidence of PONV during the entire hospitalization period was significantly lower in the CWI group compared to the groups using IV PCA (*p* < 0.05). The combined group had a significantly lower incidence of PONV and use of rescue antiemetics than the IV PCA group (*p* < 0.05). The combined group required significantly less total PCA fentanyl compared to the IV PCA group (combined vs. PCA, 622.1 μg vs. 703.1 μg, *p* < 0.001). **Conclusions**: CWI is an effective alternative to IV PCA and has fewer side effects. Combined use of CWI and IV PCA may be an ideal pain management strategy, offering a strong pain-reduction effect and only moderate side effects.

## 1. Introduction

A single-port access (SPA) laparoscopy is widely performed for the treatment of gynecologic adnexal disease [1,2]. Among minimally invasive surgeries (MISs), the SPA laparoscopy has the advantage of only one umbilical incision. However, SPA has the disadvantage of a large incision like that of a mini-laparotomy [3]. Therefore, SPA may cause more pain at the surgical site than other MISs [4,5], and an effective management strategy for surgical site pain after an SPA laparoscopy is needed. 

Pain management is one of the most important postoperative care considerations [6]. Adequate pain reduction through a combination of various pain control methods with different mechanisms of action is the goal of pain management [7,8]. Opioid-based intravenous patient-controlled analgesia (IV PCA), one of the systemic analgesia methods, has been commonly used to control postoperative pain in laparotomy and SPA laparoscopy. However, despite its strong analgesic effect, IV PCA produces several opioid-related systemic side effects such as nausea and vomiting [9]. Moreover, there are questions concerning the use of IV PCA for the SPA adnexal laparoscopy as it requires a substantially smaller wound than a laparotomy. Also, the duration of surgery is relatively shorter than for other SPA gynecologic laparoscopic procedures [10]. Continuous wound infiltration (CWI), one of the local analgesia methods, continuously injects local analgesic into the surgical site. CWI, especially in combination with a 0.5% levobupivacaine solution, has showed an effective reduction in surgical site pain without systemic side effects in a variety of surgical procedures, such as laparoscopic varicocelectomy and laparoscopic appendectomy [11,12,13]. However, the effect of CWI on reducing postoperative pain for SPA laparoscopy has not been clearly revealed due to a lack of research [14].

In this study, we compared the postoperative pain control outcomes of three pain control methods (CWI vs. IV PCA vs. CWI + IV PCA) in patients who underwent an SPA laparoscopy for adnexal disease. The purpose of this study was to determine whether CWI can replace IV PCA and to investigate effective pain control strategies after an SPA adnexal laparoscopy.

## 2. Materials and Methods

### 2.1. Study Design and Patients

We retrospectively reviewed patients who underwent an SPA laparoscopy for adnexal disease and underwent IV PCA or CWI for postoperative pain control in Kangbuk Samsung Hospital or Uijeongbu Eulji Medical Center between 1 March 2020 and 31 September 2023. For surgical inclusion criteria, only patients who underwent adnexal surgery, such as an ovarian cystectomy or salpingo-oophorectomy, were included. Those who underwent concurrent gynecologic surgery, such as a hysterectomy or myomectomy, and those who required conversion to a multiport laparoscopy or laparotomy during surgery were excluded. SPA adnexal surgery has a relatively short operation time and, unlike a laparoscopic hysterectomy, is performed only through the umbilicus without a vaginal approach. Therefore, it has relatively low possibility of occurrence of other types of postoperative pain, such as gas-induced pain, position-related pain, and surgery-related pelvic or vaginal pain, except for surgical site pain [15,16]. This is the reason we focused our study only on patients who underwent adnexal surgery.

For postoperative pain assessment, those with numeric rating scale (NRS) pain scores at 6, 12, 24, and 48 h after surgery were included. Those in whom CWI or IV PCA use was discontinued or could not be maintained over the entire hospitalization period due to side effects were excluded. Included patients were classified into one of three groups: the CWI group (CWI only), the IV PCA group (IV PCA only), and the combined group (IV PCA and CWI).

### 2.2. Ethical Aspects

This retrospective study was approved by the Institutional Review Board (IRB) of Kangbuk Samsung Hospital (IRB approval number: 2022-12-030, IRB approval date: 21 December 2023) or Uijeongbu Eulji Medical Center (IRB approval number: 2023-02-015, IRB approval date: 13 December 2023). The IRBs of both institutions waived the requirement for informed consent due to the retrospective nature of the study design.

### 2.3. Study Outcomes and Parameters

The primary outcomes were the pain control outcomes according to the three different postoperative pain management strategies (CWI vs. IV PCA vs. CWI + IV PCA). The secondary outcomes were operative outcomes and the incidence of side effects for each pain management method.

For postoperative pain outcomes, the NRS, which ranges from 0 to 10, was used to evaluate surgical site pain at 6, 12, 24, and 48 h after surgery [17]. The total amount of fentanyl citrate delivered via IV PCA over the entire hospitalization was recorded. The timing and quantity of additional pain killers, including nonsteroidal anti-inflammatory drugs (NSAIDs) or opioid drugs, were also recorded.

Patient demographic variables including age, body mass index (BMI), and history of abdominal surgery were collected. Surgical outcome-related variables were total operation time, perioperative complications, serum hemoglobin (Hb)-level change between preoperative and postoperative day 1, single-port insertion time, specimen removal time, umbilical incision length before and after surgery, duration of hospital stay (from operation day to discharge day), and pathologic diagnosis. The total operative time was defined as the duration from the initial skin incision to final skin closure. The single-port insertion time was defined as the period during which the pneumoperitoneum was maintained by CO_2_ insufflation. The specimen removal time was defined as the total time needed to extract the resected adnexal tissue through the umbilicus using the cold knife-in-bag tissue removal technique [18]. To evaluate the umbilical incision elongation caused by the elastic wound retractor of the single-port system, pneumoperitoneum, and specimen removal process during surgery, the maximum vertical length of the incision was measured both before and after surgery with the wound retractor in place [14].

We also determined the incidence of postoperative nausea and vomiting (PONV). A PONV grade from 0 to 3 was assigned to each patient (0, no nausea; 1, mild nausea; 2, severe nausea; and 3, vomiting) [19]. Oral or IV antiemetics were not administered routinely after surgery. A rescue antiemetic, metoclopramide hydrochloride, was injected intravenously if the patient experienced grade 2-or-higher PONV or if the patient asked for intervention. The frequency and total amount of administered antiemetic medication were recorded.

### 2.4. Postoperative Pain Management Protocol

The routine pain management protocol following an SPA adnexal laparoscopy was consistent at the two institutions. Before surgery, patients received detailed counseling on the clinical benefits and risks of IV PCA and CWI and then decided on a preferred method. There was no random assignment by the physicians. In the PCA group, depending on the patient age and body weight, 900–1800 μg fentanyl citrate was diluted in 100 mL of 0.9% normal saline (Table S1). The baseline infusion rate was set at 1 cc/h. When patients requested additional pain relief, a bolus dose of 15 μg fentanyl citrate was administered, with a lockout time of 15 min. In the CWI (ON-Q PainBuster^®^, I-Flow Corporation, Halyard Health, Irvine, CA, USA) group, the infusion catheter of the CWI system was positioned on the musculo-fascial layer of the umbilical wound during surgical closing at the end of surgery. The detailed application of the CWI system to the SPA laparoscopy wound was described below (Section 2.5) and in our previous study [14]. The CWI formulation contained 100 mL of 0.3% ropivacaine solution, and this solution was continuously administered at a rate of 2 mL/h. Once an oral diet was initiated, oral NSAIDs were given twice a day until discharge. In the case of NSAIDs allergy, oral acetaminophen was administered three times a day. For intense pain, NSAIDs were administered first, and opioids were administered only when necessary. Additional pain killers were administered whenever requested by the patient. Patients who underwent adnexal laparoscopic surgery were usually discharged 1 or 2 days after surgery if they met the discharge criteria based on the ERAS protocol [20,21]. The discharge criteria at both institutions are as follows: (1) no evidence of intraoperative complications, (2) stable vital signs, (3) adequately controlled pain with oral medication, (4) ability to walk without assistance, and (5) tolerance of general diet without nausea and vomiting.

### 2.5. Surgical Procedure

All surgeries were performed with an SPA laparoscopy. A 2.0-to-2.5 cm vertical incision was made in the umbilicus using the open Hasson technique. The single-port elastic wound retractor was inserted through the umbilicus, and the maximum vertical length of the umbilical incision was measured. A single multichannel cap was placed on it. A pneumoperitoneum was created using carbon dioxide (CO_2_) at 12 mmHg. Laparoscopic surgery (ovarian cystectomy or salpingo-oophorectomy) was performed in the usual manner [22]. The excised adnexal tissues were placed into an endo-pouch specimen retrieval bag, then morcellated using the cold knife-in-bag tissue removal technique and removed through the umbilical opening. To evaluate the umbilical incision elongation caused by the elastic wound retractor of the single-port system, pneumoperitoneum, and specimen removal process during surgery, the maximum vertical length of the umbilical incision was measured again after surgery with the wound retractor in place. After that, the musculo-fascial layer was sutured. In patients undergoing CWI, after suturing the musculo-fascial layer the guiding needle was inserted into the subcutaneous layer from the lower edge of the vertical umbilical incision to a point located 7~10 cm below it. The infusion catheter of the CWI system was positioned on the musculo-fascial layer of the umbilical wound under the guidance of the guiding needle. Then, the subcutaneous layer and skin were closed, ensuring that the infusion catheter was not caught in the sutures. The infusion catheter was then connected to an elastomeric pump. The detailed method of applying the CWI system to the SPA laparoscopy wound was described in our previous study [14].

### 2.6. Statistical Analysis

All statistical analyses were performed using SPSS version 25.0 (IBM SPSS Statistics for Windows, IBM Corp., Armonk, NY, USA). Normality of the data was assessed with the Shapiro–Wilk test. Data with a normal distribution are presented as mean ± standard deviation (SD), while medians (interquartile range, IQR) were used for data with a non-normal distribution. Differences among the three pain control groups were evaluated using the Kruskal–Wallis test or analysis of variance (ANOVA) for continuous variables and a multiple comparison was performed by a post hoc test using the Bonferroni method. Frequency distributions among categorical variables for the two pain control methods were compared using the chi-square test or Fisher’s exact test. A *p*-value < 0.05 was considered statistically significant.

## 3. Results

**Figure 1:**
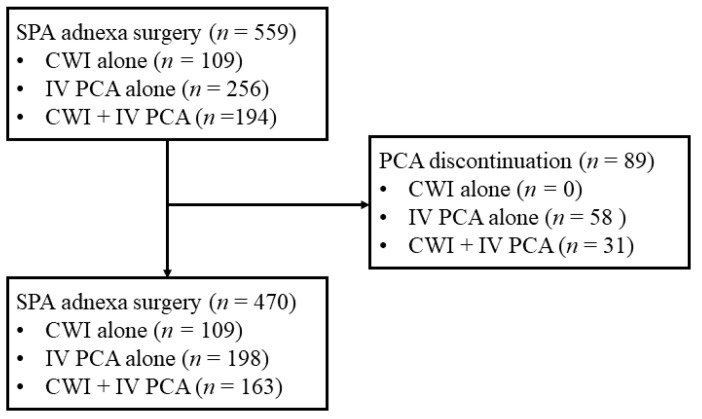
Patient selection. SPA: single-port access, CWI: continuous wound infiltration, IV PCA: intravenous patient-controlled analgesia.

During the study period, 559 patients underwent an SPA laparoscopy for adnexal disease and received CWI or IV PCA for postoperative pain management in both institutions. Of these, 89 patients who discontinued IV PCA due to side effects were excluded. No one discontinued CWI due to side effects during hospitalization. Therefore, 470 patients were included in this study. Of these, 109 (23.2%) received only CWI (CWI group), 198 (42.1%) received only IV PCA (IV PCA group), and 163 (34.7%) received both IV PCA and CWI simultaneously (combined group) (Figure 1). Table 1 presents the patient characteristics and surgical outcomes of the three groups. There were no significant differences in age, BMI, umbilical incision length before and after surgery, specimen removal time, operation time, and pathologic diagnosis among the three groups.

**Figure 2:**
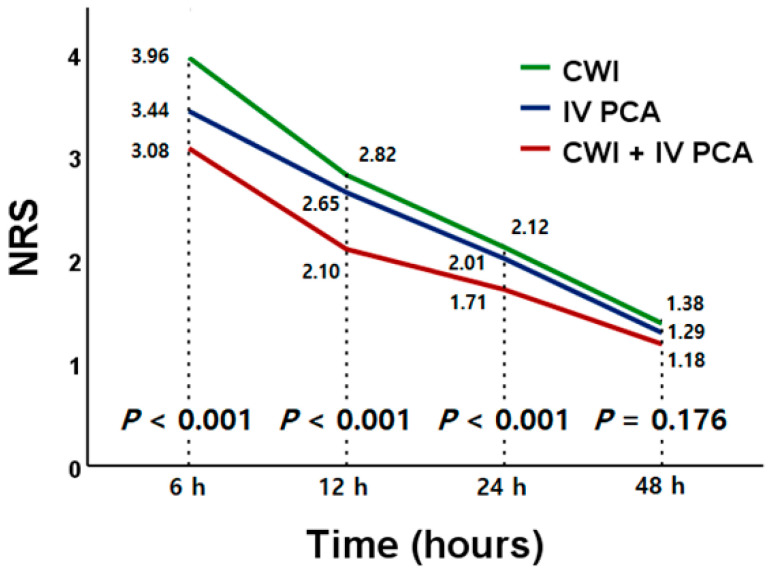
Changes in NRS pain scores over time after surgery among the three groups. NRS: numeric rating scale, CWI: continuous wound infiltration, IV PCA: intravenous patient-controlled analgesia.

The results of the postoperative surgical site pain control assessment for the three pain control methods are presented in Table 2 and Table S2. The NRS pain score tended to gradually decrease over time in all three groups (Figure 2), and the CWI group and combined treatment group showed rapid decreases in pain during the acute phase. The mean NRS scores assessed at postoperative 6 h (CWI vs. PCA vs. combined, 3.96 ± 0.89 vs. 3.44 ± 0.78 vs. 3.08 ± 0.75, *p* < 0.001), 12 h (2.82 ± 0.78 vs. 2.65 ± 0.95 vs. 2.10 ± 0.48, *p* < 0.001), and 24 h (2.12 ± 0.74 vs. 2.01 ± 0.85 vs. 1.71 ± 0.84, *p* < 0.001) were significantly lower in the combined group compared with the IV PCA group (6 h: 3.08 ± 0.75 vs. 3.44 ± 0.78, *p* < 0.001; 12 h: 2.01 ± 0.85 vs. 2.65 ± 0.95, *p* < 0.001; 24 h: 1.71 ± 0.84 vs. 2.01 ± 0.85, *p* = 0.001; Table S2) and the CWI group (6 h: 3.08 ± 0.75 vs. 3.96 ± 0.89, *p* < 0.001; 12 h: 2.01 ± 0.85 vs. 2.82 ± 0.78, *p* < 0.001; 24 h: 1.71 ± 0.84 vs. 2.12 ± 0.74, *p* < 0.001; Table S2). In particular, there was no difference in pain scores between the CWI group and the IV PCA group except for the acute postoperative period (postoperative 6 h: CWI vs. PCA, 3.96 ± 0.89 vs. 3.44 ± 0.78, *p* < 0.001; Table S2). However, after sufficient time had passed after surgery (postoperative 48 h), there was no difference in surgical site pain intensity depending on the pain control method. The total amount of fentanyl citrate injected through IV PCA during the hospitalization period was significantly less in the combined group compared to the IV PCA group (PCA vs. combined, 703.1 ± 139.1 μg vs. 622.1 ± 105.3 μg, *p* < 0.001). The proportion of patients who required rescue analgesics in addition to the routine postoperative pain control protocol was significantly lower in the combined group than in the IV PCA group (6–12 h: 12.9% vs. 21.7%, *p* = 0.029; Table S2) and the CWI group (0–6 h: 19.6% vs. 52.9%, *p* < 0.001; 6–12 h: 12.9% vs. 24.8%, *p* = 0.012; Table S2) within 12 h. When the CWI and IV PCA groups are compared, the CWI group had a higher percentage of patients requiring rescue analgesics within 6h after surgery (26.8% vs. 52.9%, *p* < 0.001; Table S2), but there was no difference in the remaining time checkpoints. Furthermore, simultaneous use of CWI and IV PCA significantly decreased the total amount of rescue analgesics required within 12 h after surgery. However, using CWI alone did not increase rescue analgesic use compared to using IV PCA alone (Table S2). The use of higher potency rescue analgesics such as tramadol hydrochloride and pethidine hydrochloride were less frequent in the combined group than the CWI and IV PCA groups, but the difference did not reach statistical significance.

The incidence of PONV of a grade 2 or higher and the requirement for rescue antiemetics for the three pain control methods are summarized in Table 3 and Table S3. Over the entire hospitalization period, nausea and vomiting occurred significantly less frequently in patients who did not receive IV PCA (the CWI group). Among patients with IV PCA (IV PCA and combined group), simultaneous use of the CWI system significantly reduced the incidence of PONV (combined vs. PCA; 0–6 h: 39.2% vs. 51.5%, *p* = 0.020; 6–12 h: 26.3% vs. 42.9%, *p* = 0.001; 12–24 h: 15.9% vs. 29.2%, *p* = 0.003; Table S3). The total amount of antiemetics used in the CWI group was also the lowest among the three groups. Adding CWI to IV PCA significantly reduced the use of antiemetics compared to using IV PCA alone (0–6 h: 0.52 ± 0.71 ampules vs. 0.73 ± 0.79 ampules, *p* = 0.011; 6–12 h: 0.25 ± 0.51 ampules vs. 0.50 ± 0.65 ampules, *p* < 0.001; 12–24 h: 0.12 ± 0.32 ampules vs. 0.26 ± 0.44 ampules, *p* = 0.003; Table S3).

## 4. Discussion

This study evaluated the efficacy of the CWI system for postoperative surgical site pain management after an SPA laparoscopy for adnexal disease. We found that CWI alone had a similar pain control effect, except for in the acute phase (within 6 h after surgery), and had significantly fewer systemic side effects compared to IV PCA alone. We also found that the combined use of CWI and IV PCA has a synergetic effect in pain control and can reduce systemic side effects through opioid sparing compared to IV PCA alone.

Reducing postoperative pain is a major concern for physicians. In the era of MIS, there has been a technical progression from conventional laparoscopy in the past, through SPA laparoscopy, to the recent advancements in vaginal natural orifice transluminal endoscopic surgery (vNOTES), all aimed at reducing postoperative pain [23,24]. Postoperative pain is associated with the length of abdominal incision and operation time [25]. SPA laparoscopy has a number of factors that make it a more vulnerable pain than other MISs. The umbilical incision size in SPA laparoscopy is the largest among MISs because all laparoscopic instruments should be inserted simultaneously through the umbilical opening. Furthermore, continuous tension from the elastic wound retractor of the single-port system and extensive wound traction during the retrieval of specimens through the umbilicus using an in-bag removal process can lead to unintended elongation of the umbilical wound. According to previous studies, depending on the characteristics of the specimen and the removal time, an unintentional umbilical incision extension of approximately 1–3 mm may occur during the in-bag tissue removal process [14,26]. Compared to other gynecologic SPA laparoscopies, such as myomectomies and hysterectomies, the SPA adnexal laparoscopy requires a short operation time [10] and specimen removal time [14,26] due to the nature of the adnexal tissue. The possibility of wound extension during an SPA adnexal laparoscopy is low [14]. Therefore, an effective but not excessive postoperative pain management strategy is needed because SPA adnexal surgery has relatively fewer factors that can worsen postoperative pain than other gynecologic SPA laparoscopic surgeries.

Appropriate postoperative pain control is an important part of postoperative management because it affects the patient’s rapid recovery and shortens the hospitalization period. The Enhanced Recovery after Surgery (ERAS) protocol also emphasizes the importance of pain management through a combination of analgesics with different mechanisms of action [27]. It is true that there has been less interest in pain management after MIS compared to a laparotomy due to its small incision. However, as the proportion of MISs increases in the gynecologic field, the importance of pain management after MIS is increasingly emphasized. Following this trend, the recent American Association of Gynecologic Laparoscopists (AAGL) guideline also reported that application of multimodal pain control methods is essential to reduce opioid usage after MIS [20]. Opioid-based IV PCA has been traditionally used for postoperative pain relief in MISs due to its convenience, short duration of action, and strong analgesic effect [28]. However, opioid-related systemic side effects such as PONV, dizziness, and hypotension can lead to premature discontinuation of IV PCA despite its powerful analgesic effect [29,30]. According to a previous study, approximately 7–20% of patients discontinued IV PCA [9,30,31]. In our study, approximately 16% of 559 patients who received IV PCA discontinued its use due to severe side effects (Figure 1). In addition, although there are differences depending on the study, the incidence of PONV in patients with fentanyl-based IV PCA has been reported to be up to approximately 80% [9,32,33,34]. In our study, approximately 61% of patients who used IV PCA alone suffered from PONV and received intervention to relieve symptoms. For this reason, the suitability of IV PCA use for MISs requiring small incisions has been questioned because of the systemic side effects. Choi et al. [31] reported that IV PCA may not be essential in certain MIS patients with colorectal disease. Yeo et al. [35] suggested that a multimodal analgesia combination (including pregabalin, tramadol, CWI, and transversus abdominis plane block) is not inferior to opioid-based IV PCA in terms of pain control and can be an effective alternative with fewer side effects. Therefore, establishing an effective pain management strategy that can replace IV PCA or reduce opioid use is essential.

Local analgesia, local blocking of pain transmission from the peripheral nerve, is a relatively safe pain control method. However, its use for postoperative pain management is limited because of the frequency of required injections due to its relatively short action time [36]. The CWI system, however, can effectively control pain through the continuous administration of local anesthetic directly into the surgical site. The ERAS group proposed that physicians should pay attention to the value of CWI as part of multimodal analgesia approaches [37]. Many previous studies focusing on large laparotomic wounds have reported that CWI is effective in reducing pain and has an opioid-sparing effect [11]. According to a study that compared CWI use only to IV PCA use only for laparotomic wounds, the two methods showed similar pain-reducing effects [38,39]. However, there have been no studies comparing CWI alone and IV PCA alone after SPA laparoscopy. In this study, we compared the pain-reduction effects of CWI alone and IV PCA alone after SPA laparoscopy for adnexal disease. CWI alone showed a similar pain control effect as IV PCA alone except for immediately after surgery (within 6 h) (Table S2) and there was no difference in the hospitalization period between the two groups. On the other hand, the incidence of PONV and the antiemetics requirement were significantly lower in the CWI-alone group than in the IV PCA-alone group (Table S2). In particular, in the acute phase within 6 h after surgery, CWI was inferior to IV PCA in pain control. Immediately after surgery, not only surgical site pain but also various types of pain related to surgery or anesthesia such as gas-induced pain, position-related pain, shoulder pain, and visceral pain could affect the pain score. We interpreted that systemic opioid-based IV PCA would be more effective in terms of pain control in the acute phase. However, the difference in PONV incidence between the CWI and IV PCA groups (24.7% vs. 51.5%) in the acute phase is still an important issue that needs to be carefully considered. In a previous study, we revealed that the combined use of CWI and IV PCA was effective for postoperative pain control and had an opioid-sparing effect in patients who underwent an SPA laparoscopy compared to IV PCA alone [14]. In the present study, simultaneous use of the CWI system and IV PCA (the combined group) showed the strongest pain-reducing effect among the three methods and significantly reduced the total amount of additional analgesia. It also significantly reduced the total quantity of opioids and the incidence of PONV compared to IV PCA alone. Our study result demonstrated that adding CWI to IV PCA maximized the pain control effect due to the synergetic effect and reduced the PONV due to the opioid-sparing effect. Considering both the analgesic effect and side effects, we suggest that the simultaneous use of IV PCA and CWI, which showed the strongest analgesic effect and moderate side effects, is the most ideal pain control strategy after SPA adnexal laparoscopy among the three methods. On the other hand, IV PCA alone is the worst method among the three methods and can be replaced by CWI alone, which has the similar pain-reduction effect and fewer side effects.

To the best of our knowledge, this is the first study to evaluate the pain-reduction effect of CWI alone for gynecologic SPA laparoscopy. However, this study has some limitations. First, this was not a prospective, randomized clinical trial. Second, there was no control group that did not use anything to control postoperative pain. Third, although various types of pain may arise following laparoscopic surgery, including gas-induced abdominal pain, shoulder pain, position-related discomfort, surgery-related pelvic or vaginal pain, and visceral pain, this study assessed only surgical site pain. Fourth, among various gynecologic SPA laparoscopies, only patients who underwent surgery for adnexal disease were included. The SPA laparoscopy for adnexal disease requires a short operation time and specimen retrieval time; other gynecologic SPA laparoscopies, such as myomectomies, hysterectomies, and endometriosis surgery, require longer operation times and specimen retrieval times, and can cause surgery-related pelvic or vaginal pain. Surgical site pain may vary between these procedures, and the role of CWI may be different according to surgery type. In conclusion, our study demonstrated that the analgesic effect of CWI alone for the SPA wound is not inferior to IV PCA except for in the acute phase. Considering that there are fewer side effects of CWI compared to IV PCA, CWI alone can be an effective alternative pain control strategy to IV PCA alone. In addition, considering both pain relief and the occurrence of side effects, we cautiously suggest that combined use of CWI and IV PCA may be an ideal pain management strategy.

## Figures and Tables

**Table 1:** Patient demographics and surgical outcomes.

Characteristics	Total	CWI	IV PCA	Combined	*p*-Value
	(n = 470)	(n = 109)	(n = 198)	(n = 163)	
Age (years)	40.19 ± 14.64	43.42 ± 15.51	39.39 ± 14.73	40.05 ± 13.74	0.258 ^a^
BMI (kg/m^2^)	23.1 ± 3.9	23.4 ± 4.2	23.1 ± 3.7	22.8 ± 3.9	0.459 ^a^
Previous OP. history, n (%)					
Laparoscopy	86 (18.3)	21 (19.3)	28 (14.1)	37 (22.7)	0.107 ^b^
Laparotomy	97 (20.6)	30 (27.5)	28 (14.1)	39 (23.9)	0.009 ^b^
Operation type, n (%)					<0.001 ^b^
Cystectomy	278 (59.1)	40 (36.7)	134 (67.7)	104 (63.8)	
Adnexectomy	192 (40.9)	69 (63.3)	64 (32.3)	59 (36.2)	
Hb change (mg/dL)	1.96 ± 0.86	1.83 ± 0.74	1.88 ± 0.91	2.13 ± 0.91	0.185 ^a^
Pre-OP. wound length (cm)	2.49 ± 0.06	2.50 ± 0.05	2.49 ± 0.06	2.49 ± 0.05	0.201 ^a^
Post-OP. wound length (cm)	2.56 ± 0.08	2.57 ± 0.08	2.56 ± 0.08	2.55 ± 0.09	0.086 ^a^
OP. time (min)	56.62 ± 18.93	55.48 ± 11.25	57.39 ± 19.54	56.62 ± 22.78	0.842 ^a^
Single-port insertion time (min)	34.73 ± 16.28	31.24 ± 9.44	35.62 ± 15.15	35.18 ± 21.54	0.347 ^a^
Specimen removal time (min)	1.58 ± 2.30	1.33 ± 0.71	1.69 ± 2.52	1.61 ± 2.69	0.410 ^a^
Hospital stay (days)	3 (3–3)	3 (3–3)	3 (2–3)	2 (2–3)	0.126 ^c^
Diagnosis, n (%)					0.089 ^b^
Teratoma	99 (21.1)	22 (20.2)	45 (22.7)	32 (19.6)	
Endometriosis	163 (34.7)	22 (20.2)	69 (34.8)	72 (44.2)	
Other benign conditions	191 (40.6)	58 (53.2)	78 (40.8)	55 (33.7)	
Borderline ovarian tumor	14 (3.0)	6 (5.5)	5 (2.5)	3 (1.8)	
Ovarian cancer	3 (0.6)	1 (0.9)	1 (0.5)	1 (0.6)	

BMI: body mass index, IV PCA: intravenous patient-controlled analgesia, CWI: continuous wound infusion, OP.: operation. Values are given as mean ± standard deviation or number (percentage). ^a^ One-way analysis of variance (ANOVA) was used. ^b^ The chi-square test was used. ^c^ The Krukal–Wallis test was used.

**Table 2:** Pain control outcomes.

Outcomes	Total	CWI	IV PCA	Combined	*p*-Value
	(n = 470)	(n = 109)	(n = 198)	(n = 163)	
NRS (mean ± SD)					
Post-OP. 6 h	3.44 ± 0.86	3.96 ± 0.89	3.44 ± 0.78	3.08 ± 0.75	<0.001 ^a^
Post-OP. 12 h	2.50 ± 0.83	2.82 ± 0.78	2.65 ± 0.95	2.10 ± 0.48	<0.001 ^a^
Post-OP. 24 h	1.93 ± 0.84	2.12 ± 0.74	2.01 ± 0.85	1.71 ± 0.84	<0.001 ^a^
Post-OP. 48 h	1.27 ±0.87	1.38 ± 0.86	1.29 ± 0.97	1.18 ± 0.74	0.176 ^a^
PCA fentanyl quantity (μg)	669.3 ± 123.7	n/a	703.1 ± 139.1	622.1 ± 105.3	<0.001 ^b^
Use of additional pain killer, n (%)					
Within 6 h	142 (30.2)	57 (52.9)	53 (26.8)	32 (19.6)	<0.001 ^c^
6–12 h	91 (19.4)	27 (24.8)	43 (21.7)	21 (12.9)	0.028 ^c^
12–24 h	60 (12.8)	16 (14.7)	26 (13.1)	18 (11.0)	0.665 ^c^
24–48 h	44 (9.4)	9 (8.3)	19 (9.6)	16 (9.8)	0.911 ^c^
Number of additional painkiller ampules used (mean ± SD)					
Within 6 h	0.53 ± 0.59	0.61 ± 0.81	0.56 ± 0.49	0.43 ± 0.52	0.024 ^a^
6–12 h	0.23 ± 0.42	0.30 ± 0.46	0.25 ± 0.43	0.17 ±0.37	0.023 ^a^
12–24 h	0.14 ± 0.38	0.17 ± 0.41	0.15 ± 0.37	0.12 ± 0.37	0.539 ^a^
24–48 h	0.10 ± 0.37	0.09 ± 0.29	0.11 ± 0.41	0.10 ± 0.36	0.916 ^a^
Type of additional painkiller used, n (%)					
NSAIDs	204 (43.4)	61 (55.9)	87 (43.9)	56 (34.3)	0.002 ^c^
Tramadol hydrochloride	55 (11.7)	16 (14.6)	25 (12.6)	14 (8.5)	0.269 ^c^
Pethidine hydrochloride	40 (8.5)	14 (12.8)	18 (9.1)	8 (4.9)	0.066 ^c^
Morphine sulfate	0	0	0	0	-

NRS: numeric rating scale, IV PCA: intravenous patient-controlled analgesia, OP.: operation, NSAIDs: nonsteroidal anti-inflammatory drugs. Values are given as mean ± standard deviation or number (percentage). ^a^ One-way analysis of variance (ANOVA) was used. ^b^ The Student’s *t*-test was used. ^c^ The chi-square test was used.

**Table 3:** The incidence of PONV and antiemetics usage.

	Total	CWI	IV PCA	Combined	*p*-Value
		(n = 109)	(n = 198)	(n = 163)	
Number of patients with PONV grade 2 or higher (n (%))					
Within 6 h	193 (41.1)	27 (24.7)	102 (51.5)	64 (39.2)	<0.001 ^a^
6–12 h	142 (30.2)	14 (12.8)	85 (42.9)	43 (26.3)	<0.001 ^a^
12–24 h	94 (20.0)	10 (9.1)	58 (29.2)	26 (15.9)	<0.001 ^a^
24–48 h	47 (10.0)	3 (2.7)	29(14.6)	15 (9.2)	0.004 ^a^
Number of antiemetics ampules used (mean + SD)					
Within 6 h	0.55 ± 0.73	0.28 ± 0.51	0.73 ± 0.79	0.52 ± 0.71	<0.001 ^b^
6–12 h	0.32 ± 0.56	0.11 ± 0.31	0.50 ± 0.65	0.25 ± 0.51	<0.001 ^b^
12–24 h	0.16 ± 0.37	0.06 ± 0.23	0.26 ± 0.44	0.12 ± 0.32	<0.001 ^b^
24–48 h	0.06 ± 0.24	0.01 ± 0.09	0.10 ± 0.31	0.05 ± 0.22	0.004 ^b^

CWI: continuous wound infiltration, IV PCA: intravenous patient-controlled analgesia, PONV: postoperative nausea and vomiting. Values are given as mean ± standard deviation or number (percentage). ^a^ The chi-square test was used. ^b^ One-way analysis of variance (ANOVA) was used.

## Data Availability

The data presented in this study are available on request from the corresponding author.

## Supplementary Materials

The following supporting information can be downloaded at https://www.mdpi.com/article/10.3390/jcm13195718/s1, Table S1: Dosage of Fentanyl Citrate According to Age and Body Weight; Table S2: Pain control outcomes; Table S3: The incidence of PONV and antiemetics usage.
