# Supplementary material for: Can a Continuous Wound Infiltration System Replace Intravenous Patient-Controlled Analgesia for Postoperative Pain Management after a Single-Port Access Laparoscopy?
Source: J Clin Med. 2024 Sep 25;13(19):5718. doi: 10.3390/jcm13195718 (PMC11477422; doi:10.3390/jcm13195718)
Supplement: Supplementary file 1 [file jcm-13-05718-s001.zip › Supplementary table.pdf]

Supplementary table S1. Dosage of Fentanyl Citrate According to Age and Body Weight**.**

**Body weight**	**Age**	
	**<70 years**	**≥70 years**
<45 kg	1200 μg	900 μg
45-59 kg	1500 μg	1200 μg
≥60 kg	1800 μg	1500 μg

Supplementary table S2. Pain control outcomes.

	CWI (*n* = 109)	IV PCA (*n* = 198)	*P*	CWI (*n* = 109)	Combined (*n* = 163)	*P*	IV PCA (*n* = 198)	Combined (*n* = 163)	*P*
NRS (mean ± SD)									
6 h	3.96 ± 0.89	3.44 ± 0.78	<0.001	3.96 ± 0.89	3.08 ± 0.75	<0.001	3.44 ± 0.78	3.08 ± 0.75	<0.001
12 h	2.82 ± 0.78	2.65 ± 0.95	0.124	2.82 ± 0.78	2.10 ±0.48	<0.001	2.65 ± 0.95	2.10 ±0.48	<0.001
24 h	2.12 ± 0.74	2.01 ± 0.85	0.261	2.12 ± 0.74	1.71 ±0.84	<0.001	2.01 ± 0.85	1.71 ±0.84	0.001
48 h	1.38 ± 0.86	1.29 ± 0.97	0.242	1.38 ±0.86	1.18 ± 0.74	0.016	1.29 ± 0.97	1.18 ± 0.74	0.237
Use of additional painkiller, n (%)									
–6 h	57 (52.9)	53 (26.8)	<0.001	57 (52.9)	32 (19.6)	<0.001	53 (26.8)	32 (19.6)	0.112
6–12 h	27 (24.8)	43 (21.7)	0.542	27 (24.8)	21 (12.9)	0.012	43 (21.7)	21 (12.9)	0.029
12–24 h	16 (14.7)	26 (13.1)	0.706	16 (14.7)	18 (11.0)	0.374	26 (13.1)	18 (11.0)	0.546
24–48 h	9 (8.3)	19 (9.6)	0.714	9 (8.3)	16 (9.8)	0.680	19 (9.6)	16 (9.8)	0.994
Number of additional painkillers ampules used (mean ± SD)									
-6 h	0.61 ± 0.81	0.56 ±0.49	0.468	0.61 ± 0.81	0.43 ± 0.52	0.022	0.56 ±0.49	0.43 ± 0.52	0.015
6-12 h	0.30 ± 0.46	0.23 ± 0.42	0.345	0.30 ± 0.46	0.17 ± 0.37	0.007	0.23 ± 0.42	0.17 ± 0.37	0.045
12-24 h	0.17 ± 0.41	0.14 ±0.38	0.541	0.17 ± 0.41	0.12 ± 0.37	0.275	0.14 ±0.38	0.12 ± 0.37	0.541
24-48 h	0.09 ± 0.29	0.10 ± 0.37	0.680	0.09 ± 0.29	0.10 ±0.36	0.779	0.10 ± 0.37	0.10 ±0.36	0.869
Type of additional painkiller used, n (%)									
NSAIDs	61 (55.9)	87 (43.9)	0.044	61 (55.9)	56 (34.3)	<0.001	87 (43.9)	56 (34.3)	0.064
Tramadol	16 (14.6)	25 (12.6)	0.613	16 (14.6)	14 (8.5)	0.116	25 (12.6)	14 (8.5)	0.219
Pethidine	14 (12.8)	18 (9.1)	0.303	14 (12.8)	8 (4.9)	0.019	18 (9.1)	8 (4.9)	0.126
Morphine	0	0	n/a	0	0	n/a	0	0	n/a

CWI; continuous wound infiltration, IV PCA; intravenous patient-controlled analgesia, NRS; numeric rating scale, NSAIDs; nonsteroidal anti-inflammatory drugs, n/a; not applicable. Values are given as mean ± standard deviation or number (percentage).

$^{*}P < 0.05$

Supplementary table S3. PONV

	CWI (n = 109)	IV PCA (n = 198)	*P*	CWI (n = 109)	Combined (n = 163)	*P*	IV PCA (n = 198)	Combined (n = 163)	*P*
Number of patients with PONV grade 2 or higher, n (%)									
–6h	27 (24.7)	102 (51.5)	<0.001	27 (24.7)	64 (39.2)	0.013	102 (51.5)	64 (39.2)	0.020
6–12 h	14 (12.8)	85 (42.9)	<0.001	14 (12.8)	43 (26.3)	0.007	85 (42.9)	43 (26.3)	0.001
12–24 h	10 (9.1)	58 (29.2)	<0.001	10 (9.1)	26 (15.9)	0.106	58 (29.2)	26 (15.9)	0.003
24–48 h	3 (2.7)	29 (14.6)	0.001	3 (2.7)	15 (9.2)	0.036	29 (14.6)	15 (9.2)	0.116
Number of antiemetics ampules used (mean ± SD)									
–6 h	0.28 ± 0.51	0.73 ± 0.79	<0.001	0.28 ± 0.51	0.52 ± 0.71	0.002	0.73 ± 0.79	0.52 ± 0.71	0.011
6–12 h	0.11 ± 0.31	0.50 ± 0.65	<0.001	0.11 ± 0.31	0.25 ± 0.51	0.009	0.50 ± 0.65	0.25 ± 0.51	<0.001
12–24 h	0.06 ± 0.23	0.26 ± 0.44	<0.001	0.06 ± 0.23	0.12 ± 0.32	0.086	0.26 ± 0.44	0.12 ± 0.32	<0.001
24–48 h	0.01 ± 0.09	0.10 ± 0.31	0.002	0.01 ± 0.09	0.05 ± 0.22	0.072	0.10 ± 0.31	0.05 ± 0.22	0.067

CWI; continuous wound infiltration, IV PCA; intravenous patient-controlled analgesia, PONV; postoperative nausea and vomiting. Values are given as mean ± standard deviation or number (percentage).

* $P < 0.05$
